# Complete solubilization of mammalian cells in lysates

**DOI:** 10.1016/j.mex.2024.102860

**Published:** 2024-07-11

**Authors:** Izabela Bednarska, Darina Malycheva, Maria Alvarado Kristensson

**Affiliations:** Molecular Pathology, Department of Translational Medicine, Lund University, Inga Marie Nilssons gata 53, SE-214 28 Malmö, Sweden

**Keywords:** Mammalian cells, Cell debris, Protein solubilization, Whole cells, Western blotting, Whole cell lysates preparation

## Abstract

In conventional cell lysate protocols, cell debris is typically discarded to obtain a cleaner lysate. However, this approach has limitations, as it may overlook vital cellular components. By discarding cell debris, researchers may inadvertently exclude crucial elements. Retaining all cellular components offers several advantages for studying molecular biology within various cellular compartments. Firstly, it provides a more accurate representation of the cellular environment. Secondly, it enables the study of complex cellular interactions, including those involving cellular structures and signaling pathways associated with debris. This shift in perspective highlights the importance of a holistic approach to lysate preparation. By obtaining lysates that include all cellular components, researchers can gain deeper insights into cellular processes, leading to more accurate data and a better understanding of cellular function and dysfunction. This study aimed to develop a protocol for the preparation of total cell lysates that retain all cellular components, including debris. Our method involves:

• A three-step solubilization process using a combination of detergents, saccharides, and chelators, coupled with sonication, in contrast to the classical one-step approach using an all-detergent cocktail.
• A comprehensive strategy ensuring the solubilization of all cellular components, providing a more complete lysate for analysis.

## Graphical abstract

**Figure:**
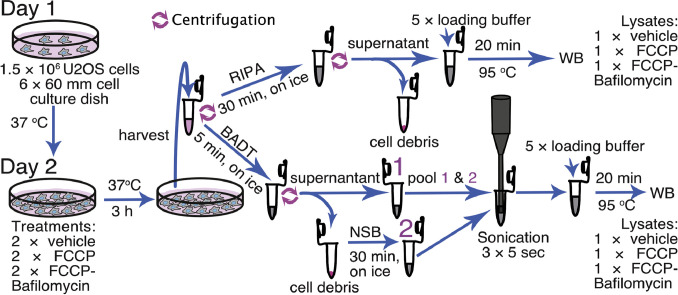

Specifications table

**Table:** 

Subject area:	Biochemistry, Genetics and Molecular Biology
More specific subject area:	Detection of the level of expression of proteins in a cell extract
Name of your method:	Whole cell lysates preparation
Name and reference of original method:	https://doi.org/10.1038/s42003–021–02280–1
Resource availability:	NA

## Protocol background

The preparation of cell lysates is a fundamental step in various analytical techniques, such as Western Blotting (WB). Traditionally, cell lysate preparation involves the removal of cell debris to achieve a cleaner sample for downstream applications. However, this conventional approach can inadvertently exclude essential cellular components, potentially skewing the understanding of cellular processes and protein interactions.

The RIPA (Radio-Immunoprecipitation Assay) buffer is commonly used for rapidly lysing cells due to its ability to effectively solubilize proteins from various cellular compartments. This effectiveness is achieved through a detergent blend composed of NP-40, sodium deoxycholate, and sodium dodecyl sulfate (SDS) [1]. In the RIPA method, cells are lysed, and the lysate is then centrifuged to remove the remaining cell debris. This process, however, results in the loss of critical elements associated with non-soluble fractions. The exclusion of these components can limit the comprehensiveness of the analysis, highlighting the need for protocols that retain all cellular elements, including debris.

Our study aims to address this gap by developing a comprehensive cell lysis protocol that preserves all cellular components. The developed protocol consists of three-steps [[2], [3], [4], [5]]. In the first step, cytosolic proteins are solubilized with triton X-100 in an isotonic buffer supplemented with Na_3_VO_4_ [6], ensuring the preservation of phosphorylated proteins, cellular integrity and prevents osmotic shock of organelles. In the second step, the lysate is centrifuged, and the supernatant is separated from the pellet. The pellet, which contains non-soluble fractions, is then solubilized in a non-salt buffer (NSB) [6]. In the final step, the pooled fractions (supernatant and solubilized pellet) are further solubilized using sonication. This step enhances the solubilization of cellular components, providing a more complete lysate for analysis.

This method significantly improves the solubilization and retention of cellular components, offering a more comprehensive lysate compared to traditional RIPA buffer protocols. By retaining all cellular elements, our protocol enables a more accurate study of cellular processes and protein interactions. However, it is important to note that, in comparison to RIPA, our procedure is more time-consuming and complex.

## Method details

Cell lysates preparation for Western Blot analysis

*Step 1: preparation of cell lysates*

Materials

• U2OS human osteosarcoma cells (ATCC®HTB-96™)
• 60-mm tissue culture dishes
• Carbonyl cyanide 4-(trifluoromethoxy)phenylhydrazone (FCCP; Sigma-Aldrich-Merck, cat. no. C2920): an uncoupler of oxidative phosphorylation in mitochondria [7] that impairs protein degradation due to decreased ATP levels, affecting both proteasomal and autophagic pathways [8]
• Bafilomycin A1 (MedChemExpress, cat. No. AB HY-100558): a reversible inhibitor of vacuolar *H*^+^-ATPase (V-ATPase) that blocks autophagosome-lysosome fusion, leading to the accumulation of autophagosomes and their protein contents [9]
• Phosphate buffered saline (PBS; Medicago, cat. no. M09–9400–100): 137 mM NaCl, 2.7 mM KCl, 10 mM Na2HPO4, 1.8 mM KH2PO4, and deionized distilled water
• Trypsin 0.25 % (Hyclone, cat. no. SH30042.01)
• RIPA buffer: freshly made 50 mM Tris–HCl pH7.5 (BioChemica, cat. no. T1513.1000), 1 % NP-40 (BioChemica, cat. no. A1694,0250), 0.5 % sodium deoxycholate (Sigma-Aldrich-Merck, cat. no. 30970), 0.1 % (w/v) sodium dodecyl sulfate (SDS; Sigma-Aldrich, cat. no. GE17–1313–01), 150 mM NaCl, 200 mM phenymethanesulfonyl fluoride (PMSF; Sigma-Aldrich-Merck, cat. no. PMSF-RO), and 1 mM dithiothreitol (DTT; Sigma-Aldrich-Merck, cat. no. D0632) dissolved in deionized water
• BA buffer, which can be stored at –20 °C for several months: freshly made 10 mM Hepes pH 7.9 (GE Healthcare, cat. no. SH30237.01), 10 mM KCl, 10 % glycerol (v/v; Sigma- Aldrich, cat. no. G5516), 1.5 mM MgCl_2_, and 340 mM sucrose dissolved in deionized water
• BADT buffer: freshly add to the BA buffer 0.1 % triton X-100 (v/v; Sigma-Aldrich-Merck, cat. no. T8787), 1 mM DTT, 0.2 mM PMSF, and 0.1 mM Na_3_VO_4_ (Sigma-Aldrich-Merck, cat. no. T9284)
• 10X none salt buffer (NSB): 3 mM ethylenediaminetetraacetic acid tetrasodium salt dihydrate (EDTA; Scahrlau, cat. no. AC0965), and 0.2 mM Ethyleneglycol- *bis*(β-aminoethyl)-N,N,Nʹ,Nʹ-tetraacetic Acid (EGTA; AppliChem A0878,0100) dissolved in deionize water. The final 1X NSB is supplemented freshly with 1 mM DTT, 0.2 mM PMSF
• 5X loading buffer: 625 mM TRIS pH 6.5 (Sigma-Aldrich, cat. no. 10812846001), 10 % (w/v) SDS, 25 % (v/v) glycerol, 0.005 % (w/v) bromophenol blue (Sigma-Aldrich, cat. no. GE17–1329–01), 250 mM DTT, and deionized distilled water
• Phosphate buffered saline with Tween (PBS-T; Medicago, cat. no. M09–9410–100): 137 mM NaCl, 2.7 mM KCl, 10 mM Na2HPO4, 1.8 mM KH2PO4, 0.1 % (v/v) Tween 20, and deionized distilled water
• One of the following primary antibodies dissolved in PBS-T: anti-γ-tubulin (1:1000; Sigma-Aldrich-Merck, cat. no T3320), anti-LC3 (1:1000; Sigma-Aldrich-Merck, cat. no L7543), anti-histone 3 (1:1000; Santa Cruz Biotechnology, cat. no sc-517576), anti-ubiquitin (1:400; Santa Cruz Biotechnology, cat. no sc-166553), anti-actin (1:500; Santa Cruz Biotechnology, cat. no sc-47778), or anti-SLC25A6 (1:1000; Proteintech, cat. no 51031–1AP)
• One of the following secondary antibodies dissolved in PBS-T: Mouse IgG horse radish peroxidase (HRP)-linked F(ab’)2 Fragment (1:5000; Sigma-Aldrich-Merck, cat. no NA9310V) or anti-rabbit IgG HRP linked whole Ab (1:5000; Sigma-Aldrich-Merck, cat. no GENA934)
• 1.5 ml microcentrifuge tubes (Sigma-Aldrich-Merck, cat. no. Z336777)
• Soniprep 150 Plus with exponential probe (240 V; MSE, cat. no. MSS150.CX4.5)
• Acrylamide solution (40 %)– mix 37.5:1, molecular biology grade (Applichem, cat. no. A4989,0500)
• 0.22 µm nitrocellulose membrane (Advansta)
• Protein Electrophoresis system (Bio-Rad)
• Semi-Dry TransBlot Turbo Transfer System (Bio-Rad)
• 5 % (w/v) bovine serum albumin (BSA; Sigma-Aldrich-Merck, cat. no. A5611) blocking buffer (for saturating excess protein-binding sites on membranes): 2.5 g BSA and 50 ml PBS-T
• WesternBright Sirius HRP-substrate (HRP-substrate; Advansta, cat. no. K-12043)
• ChemiDoc Imaging system (CDC camera; Bio-rad)
• ReBlot Plus Strong Antibody Stripping Solution, 10X (Merck, cat. no. 2504)
• Densitometry analyses using ImageJ (Fiji) software

Note that the preceding list includes only essential cell lines and non-standard laboratory equipment.

1. Plate 1.5 × 10^6^ U2OS cells per plate in six 60 mm tissue culture dishes per independent experiment according to the instructions of the cell distributors (Fig. 1).

**Fig. 1:**
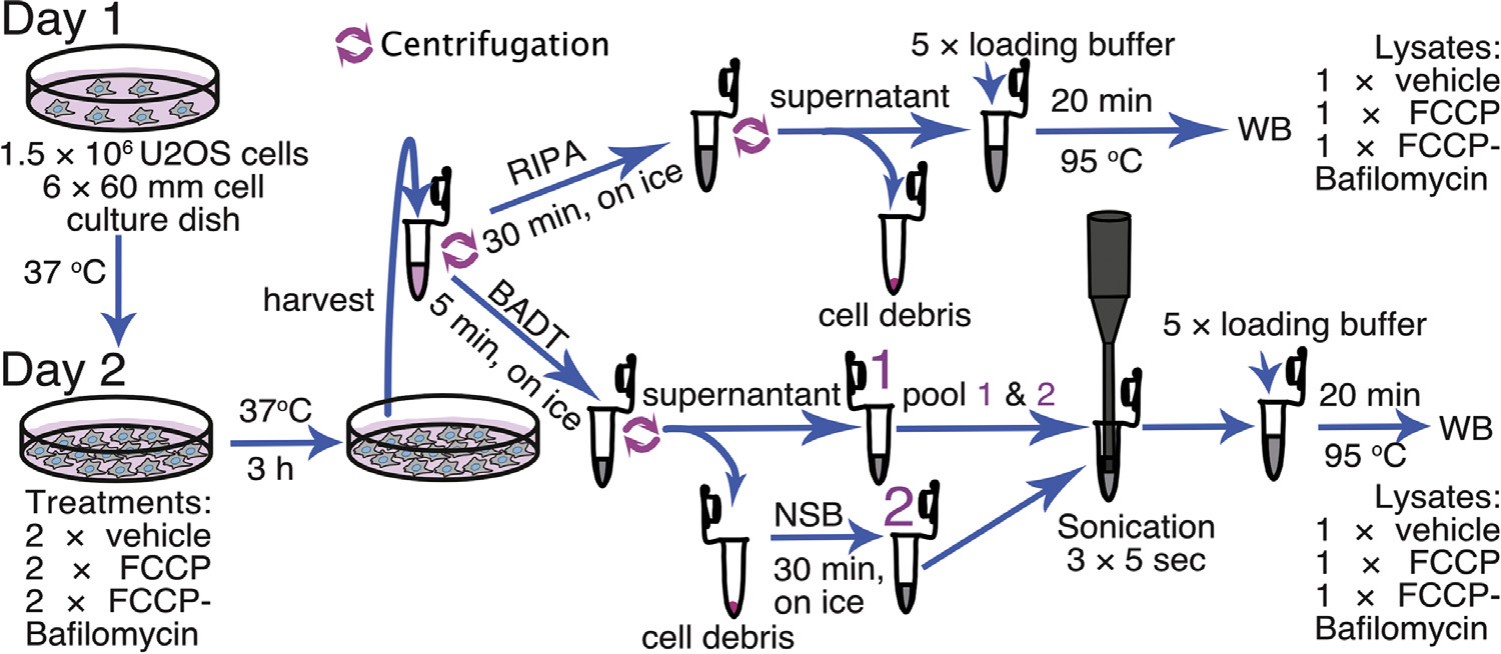
Preparation of cell lysates: a scheme illustrating the process of preparation cell lysates. In short, U2OS cells are cultured overnight. The next day, the cells are treated with either the vehicle, FCCP, or a combination of FCCP and bafilomycin A1 for 3 h in the cell incubator (37 °C). Then, the cells are harvested, centrifuged, and the resulting pellets are resuspended either in RIPA or BADT buffer. The three samples resuspended in RIPA are incubated on ice for 30 min. Following this, cell debris is removed by centrifugation and discarded. The remaining supernatants are then transferred to new tubes and mixed with 5X loading buffer before heating the resulting lysates at 95 °C for 20 min. As for the three samples resuspended in BADT, they are incubated on ice for 5 min. Subsequently, cell debris is pelleted by centrifugation, and the supernatants are transferred to new tubes (labeled as 1). The remaining pellets are resuspended in non-salt buffer (NSB; labeled as 2) and incubated on ice for 30 min. Thereafter, pool together the correspondent supernatant 1 and the NSB-lysate 2. Further solubilize the resulting BADT/NSB-lysates using three sonication cycles of 5 s each. Finally, add 5X loading buffer to the resulting lysates before heating them at 95 °C for 20 min.
2. One day after plating, prepare 2.2 ml of cell medium containing either vehicle (Dimethyl Sulfoxide, control), 20 µM of FCCP, or a combination of 20 µM of FCCP and 50 nM bafilomycin A1
3. Remove the dishes from the incubator, discard the overnight medium, and then add one milliliter of the prepared medium to treat two plates of the six with either the vehicle, 20 µM of FCCP, or the combination of 20 µM of FCCP and 50 nM bafilomycin A1 (Fig. 1).
4. Incubate the dishes for 3 h in the cell incubator (Fig. 1).
5. Prepare/thaw RIPA and BADT buffer.
6. Place the buffers on ice.
7. After incubation, remove the cell medium from the plates.
8. Wash the cells with 2 ml of PBS to remove any remaining cell medium, which contains proteins that inhibit the activity of the protease trypsin. Trypsin is used to break down the proteins that bind the cells to the bottom of the plate.
9. Remove the PBS from the plate.
10. Repeat the washing process one more time.
11. Add 0.5 ml trypsin.
12. Place the plates in the incubator (37 °C, 5 % CO_2_) until the cells detach from the bottom of the plates (the trypsin solution will become cloudy). This takes a few minutes.
13. Stop the trypsin activity by adding 2 ml of fresh cell medium (which inhibits trypsin).
14. Harvest the cells into labelled tubes and pellet them by centrifugation (500 × g for 5 min at 4 °C).
15. Discard the supernatant.
16. Keep the tubes on ice.
17. Resuspend one of the pellets resulting from each treatment (vehicle, FCCP, and FCCP/ Bafilomycin A1) from a 60 mm tissue culture dish in either 150 µl RIPA or 75 µl BADT buffer by resuspending several times up and down with a pipette tip until no cell clumps are observed.
18. Place the RIPA- or BADT-resuspended cells on ice for 30 or 5 min, respectively. Occasionally, vortex the tubes briefly to increase solubilization (Fig. 1).
19. After 5 min incubation, pellet the BADT-insoluble material by centrifugation (1300 × g for 5 min at 4 °C).
20. Transfer the supernatant to new labelled tubes and resuspend each resulting pellet in 75 µl 1X NSB (Fig. 1).
21. Resuspend the three pellets by resuspending several times up and done with a pipette tip until a clear solution is obtained.
22. Place the tubes containing the NSB-resuspended debris on ice 30 min. Occasionally vortex the tubes briefly to increase solubilization.
23. After 30 min of incubation, pellet the RIPA-insoluble material by centrifugation (10,000 × g for 15 min at 4 °C).
24. Transfer the supernatant from the RIPA-solubilized cells to new labeled tubes containing 38 µl 5X loading buffer (Fig. 1).
25. Boil the three RIPA-solubilized samples at 95 °C for 20 min. If proteolysis of desired proteins is observed, shorten the incubation to 5 min or test other mild conditions (e.g., 75 °C for 10–15 min).
26. After 30 min incubation, transfer the three BADT fractions back to the corresponding tube containing the NSB-solubilized debris (Fig. 1). Vortex the resulting three BADT/NSB-lysates.
27. Further solubilize the three resulting BADT/NSB-lysates using three sonication cycles of 5 s each with 3-seconds pause between cycles, at an amplitude of 10 (Fig. 1).
28. Add 38 µl of 5X loading buffer to each BADT/NSB-lysate.
29. Boil the three BADT/NSB-solubilized samples at 95 °C for 20 min (Fig. 1), to aid further protein solubilization. If proteolysis of desired proteins is observed, shorten the incubation to 5 min or test other mild conditions (e.g., 75 °C for 10–15 min).
30. Analyze 30 µl of each sample using a 15 % acrylamide gel followed by WB [10] and transfer to a 0.22 µm nitrocellulose membrane according to the manufacturer's instructions.
31. After transferring the proteins to the nitrocellulose membrane, wash the membrane three times for 5 min with PBS-T before blocking with BSA blocking buffer for 1 hour at room temperature on an orbital shaker.
32. Add one of the primary antibodies and incubate at 4 °C overnight on an orbital shaker.
33. The next day, wash the membrane three times for 5 min with PBS-T.
34. Add a secondary antibody and incubate for 1 h at room temperature on an orbital shaker.
35. Develop the membrane using HRP substrate in a CDC camera according to the manufacturer's instructions.
36. Strip the membrane with 1X stripping Solution according to the manufacturer's instructions before incubation with the next primary antibody.
37. Wash the membrane twice for 5 min each with PBS-T.
38. Repeat the incubation with each primary antibody at 4 °C overnight on an orbital shaker, followed by the subsequent steps of washing, secondary antibody incubation, and development.
39. Analyze the resulting images using Fiji software.
40. Open the image of interest.
41. Convert the image to 8-bit (Image > Type > 8-bit).
42. Use the rectangle tool to select the area containing the strongest band in the WB.
43. Under the analyze menu, choose the Measure tool.
44. A result table will appear, displaying the mean value of the intensity of the selected band.
45. Move the rectangle to an area that represents the background intensity within the WB.
46. Under the analyze menu, choose the Measure tool again, and the mean value of the background intensity will be added to the results table.
47. Under the analyze menu, choose the calibrate tool, and a calibration menu will pop up with the mean values present in your results table.
48. Choose the straight Line function and set the unit to Gray Value.
49. Set the obtained mean value from the band with the highest intensity as 1 and the lowest intensity as 0 in the menu, then click OK.
50. Move the existing rectangle to the bands of interest and choose the Measure tool. Repeat this step for all the bands of interest. The mean value of each band will appear in the results table. Note that after calibration, the values of the bands will be between 1 and 0.

**Fig. 2:**
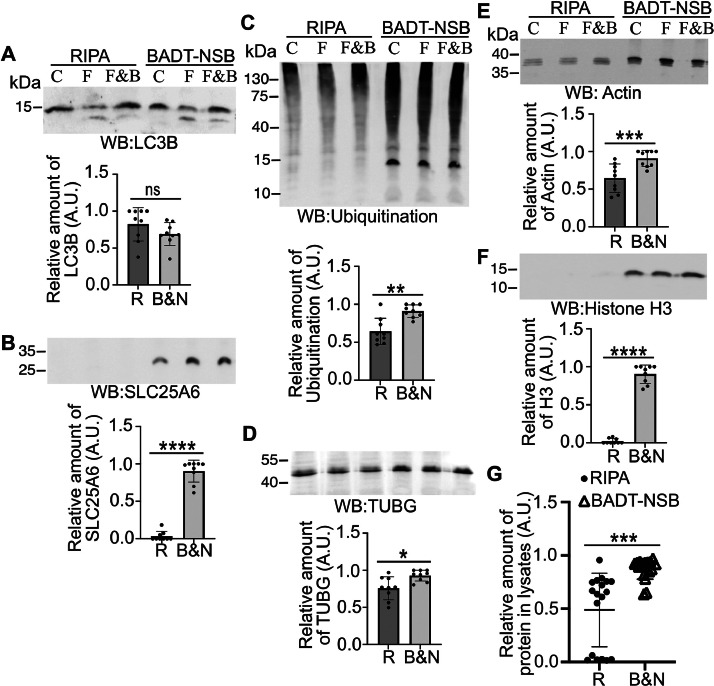
Depletion of cell debris underestimates the expression levels of proteins in total cell lysates. Total lysates of U2OS cells treated for 3 h with vehicle (C), 20 µM FCCP (F), or 20 µM FCCP and 50 nM Bafilomycin A1 (F&B) were prepared using either RIPA (R) or BADT/NSB (B&N) buffers. The protein content of LC3B (A), SLC25A6 (B), ubiquitination (C), TUBG (D), actin (E), and histone H3 (F) was analyzed by Western Blotting (WB) using an anti-LC3B (A), anti-SLC25A6 (B), anti-ubiquitination (C), anti-TUBG (D), anti-actin (E), and anti-histone H3 (H3; F) antibodies, as indicated. The graphs (A–G) illustrate the densitometric analysis of the total protein content for each treatment in the presented Western blots (G) or in the Western blot above the graph, as indicated (mean ±SD; *N* = 3, ∗*P<* 0.05, ∗∗*P<* 0.01, ∗∗∗ *P<* 0.001, ∗∗∗∗ *P<* 0.0001; ns = non-significant) from three independent experiments. (G) The graph compares the total protein content from all the proteins analyzed obtained with the different solubilizing protocols. The relative protein content was determined by setting the band with the highest protein content to one and the background as zero, with other bands being relatively calculated.

Note that most significant differences, with the exception of the microtubule-associated protein LC3B (Fig. 2A), are observed in the mitochondrial marker SLC25A6 (Fig. 2B), ubiquitinated proteins (Fig. 2C), the nuclear and cytosolic proteins TUBG (Fig. 2D) and actin (Fig. 2E), and the nuclear protein histone H3 (Fig. 2F). Compared to cells lysates prepared with RIPA, the three-step lysis method using BADT and NSB followed by sonication (Fig. 1, Fig. 2) proves to be the most effective in solubilizing cellular components. The improvement of solubilization may be attributed to the three-step procedure's ability to solubilize membrane proteins and offer difficult-to-solubilize proteins from the interior of organelles at each step. Supporting this notion, LC3B turnover does not differ between RIPA and BADT-NSB (Fig. 2A), demonstrating that our protocol allows for the detection of more proteins without altering the cellular state.

## Additional information

In conclusion, the findings highlight the superior efficacy of the three-step lysis method employing BADT and NSB followed by sonication, as evidenced by its remarkable ability to solubilize cellular components, particularly in the cases of SLC25A6, histone H3, and ubiquitinated proteins. Significant differences were also noted in TUBG and actin levels.

## Declaration of generative AI and AI-assisted technologies in the writing process

During the preparation of this work the author(s) used ChatGPT in order to improve the language. After using this tool/service, the author(s) reviewed and edited the content as needed and take(s) full responsibility for the content of the publication.

## CRediT authorship contribution statement

**Izabela Bednarska:** Validation, Writing – review & editing. **Darina Malycheva:** Supervision, Writing – review & editing. **Maria Alvarado Kristensson:** Supervision, Conceptualization, Methodology, Writing – review & editing.

## Declaration of competing interest

The authors declare that they have no known competing financial interests or personal relationships that could have appeared to influence the work reported in this paper.

## Data Availability

Data will be made available on request.
